# Is There a Gender Difference in Terms of Inflammatory Biomarkers in Patients With Severe Covid-19 Pneumonia?

**DOI:** 10.7759/cureus.32541

**Published:** 2022-12-15

**Authors:** Esma Sevil Akkurt, Tugce Sahin Ozdemirel, Ozlem Ertan, Egemen Unal, Berna Akıncı Özyürek

**Affiliations:** 1 Pulmonary Medicine, Ankara Atatürk Sanatorium Training and Research Hospital, Ankara, TUR; 2 Pulmonary Medicine, Ankara Ataturk Sanatorium Training and Research Hospital, Ankara, TUR; 3 Public Health, Ankara Yıldırım Beyazıt University Faculty of Medicine, Ankara, TUR

**Keywords:** biomarkers, inflammatory, gender, pneumonia, covid-19

## Abstract

Background

The men infected with COVID-19 have been shown to have more severe disease and a higher mortality rate. Morbidity and mortality associated with COVID-19 are mediated through intense viral inflammation and increased levels of inflammatory biomarkers. We aimed to retrospectively evaluate any gender difference in patients with severe COVID-19 pneumonia in terms of inflammatory biomarkers.

Methods

Our study included 132 patients. The general characteristics, radiological features and laboratory parameters of the patients were recorded.

Results

No difference was observed between the genders according to comorbidities, pulse steroid requirement and hypoxemia. There was no difference between the male and female participants in terms of age, white blood cell count, lymphocyte count, red cell distribution width, C-reactive protein, troponin, albumin and D-dimer. However, duration of hospitalization; percentage of polymorphonuclear leukocyte (PNL); and haemoglobin, alanine aminotransferase and ferritin values were higher in the males, and lymphocyte percentage and platelet count were higher in the women participants.

Conclusion

Larger studies with gender-specific reporting and robust analyses are required to clarify how gender alters the cellular and molecular pathways associated with COVID-19. This would improve the interpretation of biomarkers and the clinical management of COVID-19 patients by facilitating a personalised medical approach to risk stratification, prevention and treatment.

## Introduction

In January 2020, a newly identified SARS-CoV-2 agent was defined as coronavirus disease 2019 (COVID-19) after a pneumonia epidemic in the city of Wuhan, China. While COVID-19 pneumonia can result in acute respiratory distress syndrome and death, most cases have mild symptoms and a good prognosis however; men infected with SARS-CoV-2 have been shown to have more severe disease and a higher mortality rate [1]. Since no effective treatment has been developed, it is imperative to identify markers that follow the progression of COVID-19 disease and treat patients early [2]. Morbidity and mortality associated with COVID-19 are mediated through intense viral inflammation and increased levels of inflammatory biomarkers and cytokines, often referred to as a cytokine storm. It has been shown that those with an excessive inflammatory profile are mostly elderly and male patients [3,4]. We aimed to retrospectively evaluate any gender difference in patients with severe COVID-19 pneumonia in terms of inflammatory biomarkers.

## Materials and methods

Study population

We performed a single-center retrospective research. Our study included 142 patients followed up in the COVID-19 service of our hospital between December 2020 and January 2021. Ten of them were excluded because of missing data. As a result, the study included 132 patients who fully met the criteria. The participants in the study were divided into two groups as male and female.

Data collection

The clinical characteristics (age, gender, comorbidities, duration of hospital stay, pulse steroid requirement and oxygen saturation), the laboratory data of the patients (hemogram values, C-reactive protein, D-dimer, troponin and ferritin) and thorax computed tomography (CT) images were obtained retrospectively from the hospital’s data automation system and written medical records. CT examinations were performed on patients on the admission day with two multi-detector CT scanners (Emotion 6, Siemens, Germany and Alexion 16, Toshiba Medical Systems, Japan). CT images were independently reviewed by two experienced radiologists. Ground glass opacity and consolidation findings were recorded.

C-reactive protein (CRP) was measured by an immunoturbidimetric method in autoanalyzer Beckman Coulter AU580 (Beckman Coulter, Brea, CA, USA). D-dimer was measured by latex agglutination method in Diagon CoagXL (Sysmex CS 2500, Japan) device. Hemogram parameters were measured using the Impedance method on Mindray BC-6800 (Mindray Bio-Medical Electronics Co., Ltd., Shenzhen, China) auto hematology analyzer. The serum level of troponin was analyzed with the immunoassay analyzer.

The study protocol was approved by the Local Ethics Committee of our institute and was conducted according to the Declaration of Helsinki (decision no: 711, date: 01/2021).

Statistical analysis

When carrying out data analysis, an independent paired t-test (Student's t-test) was used for the comparison of the two groups, Mann-Whitney U test was used when preconditions were not available. Chi-square test was used for the analysis of categorical data. P values of ≤0.05 were accepted as statistically significant.

## Results

Of the 132 participants, 79 were male (59.8%), and the mean age of the group was 62.83 ± 14.62 years. An evaluation of the participants’ general clinical characteristics according to gender is given in Table 1.

**Table 1:** Evaluation of the participants according to the general focus

		Sex				
		Male (n=79)		Female (n=53)		
		N	%	N	%	p*
Chronic obstructive pulmonary disease	No	62	55.9	49	44.1	0.031
	Yes	17	81.0	4	19	
Asthma	No	76	63.9	43	36.1	0.004
	Yes	3	23.1	10	76.9	
Hypertension	No	51	72.9	19	27.1	0.001
	Yes	28	45.2	34	54.8	
Diabetes mellitus	No	53	60.2	35	39.8	0.900
	Yes	26	59.1	18	40.9	
Hypothyroidism	No	76	61.3	48	38.7	0.183
	Yes	3	37.5	5	62.5	
Malignancy	No	68	58.6	48	41.4	0.438
	Yes	11	68.8	5	31.3	
Rheumatological disease	No	77	61.1	49	38.9	0.175
	Yes	2	33.3	4	66.7	
Cardiac disease	No	60	60.6	39	39.4	0.758
	Yes	19	57.6	14	42.4	
Oxygen desaturation	No	14	51.9	13	48.1	0.342
	Yes	65	61.9	40	38.1	

While no difference was observed between the genders according to comorbidities, such as diabetes mellitus, hypothyroidism, malignancy, rheumatological disease, cardiac disease, pulse steroid requirement and oxygen desaturation status (p > 0.05, respectively), hypertension (p = 0.031) and asthma (p = 0.004) diagnoses were found to be more common in women, and the diagnosis of chronic obstructive pulmonary disease (COPD) (p = 0.001) was found to be more common in males. An evaluation of the participants’ laboratory values by gender is given in Table 2.

**Table 2:** Evaluation of some laboratory values of the participants according to gender RDW: Red cell distribution width; AST: Aspartate aminotransferase; ALT: Alanine aminotransferase; CRP: C-reactive protein; PNL: Polymorphonuclear leukocyte.

	Sex										
	Male					Female					
	Mean	SS		Min	Max	Mean	SS		Min	Max	p*
Age	62.49	14.04		32.00	93.00	63.34	15.58		19.00	94.00	0.563
Duration of hospitalization	13.14	8.18		2.00	52.00	10.72	7.91		0.00	40.00	0.020
Leukocyte	9197.47	4495.38		1110.00	23400.00	8120.75	3620.11		3160.00	21420.00	0.157
Lymphocyte	1117.57	648.83		90.00	3570.00	1251.34	631.80		171.00	3270.00	0.166
Lymphocyte %	14.47	9.15		0.80	45.60	18.27	10.36		2.20	46.40	0.034
PNL %	79.35	11.06		47.90	98.10	75.16	11.99		46.50	95.60	0.037
Haemoglobin	14.03	1.51		9.40	17.20	12.63	1.63		8.70	19.20	<0.001
Platelet	241.67	89.32		106.00	492.00	280.15	113.79		46.00	625.00	0.050
RDW **	14.01	1.79		11.80	24.10	14.13	2.25		12.20	23.20	0.552
ALT **	39.10	34.95		6.00	225.00	28.94	25.87		3.00	132.00	0.020
AST **	44.71	36.18		11.00	226.00	37.91	28.34		10.00	179.00	0.246
CRP **	113.12	86.98		6.95	443.00	97.46	89.34		0.68	435.00	0.172
Troponin	22.80	52.94		2.50	331.71	18.39	48.36		2.50	347.89	0.099
Albumin	32.22	5.56		14.70	44.70	33.99	5.24		22.30	43.80	0.088
Ferritin	579.77	508.85		18.30	1650.00	315.62	341.88		3.30	1650.00	0.001
D-Dimer	3.23	9.86		0.19	80.00	1.92	3.32		0.19	16.58	0.996

There was no difference between the male and female participants in terms of age, white blood cell count, lymphocyte count, red cell distribution width (RDW), aspartate aminotransferase (AST), C-reactive protein (CRP), troponin, albumin and D-dimer values (p > 0.05, respectively). However, duration of hospital stay; percentage of polymorphonuclear leukocyte (PNL); and haemoglobin, alanine aminotransferase (ALT) and ferritin values were higher in the male participants, and lymphocyte percentage and platelet count were higher in the women participants (p < 0.05, respectively). An evaluation of the participants’ radiological involvement according to gender is given in Table 3. No statistically significant difference was found in terms of ground glass appearance, consolidation and percentage of radiological involvement in thorax computed tomography for both genders (p > 0.05, respectively).

**Table 3:** Evaluation of some findings regarding the radiological involvement of the participants by gender

		Sex				
		Male		Female		
		N	%	N	%	p*
Ground glass opacity	No	4	57.1	3	42.9	0.881
	Yes	75	60.0	50	40.0	
Consolidation	No	24	60.0	16	40.0	0.981
	Yes	55	59.8	37	40.2	
Percent lung involvement (50%)		44	59.5	30	40.5	0.918
	>50%	35	60.3	23	39.7	

## Discussion

It has been shown that men are at higher risk than women for serious COVID-19 infection. Male individuals account for approximately 60% of deaths attributed to COVID-19 [5]. Gender differences have been described for many inflammatory markers, including CRP and IL-6 [6-8]. Despite the growing body of evidence that supports a sex difference in immune response, it is unknown how inflammation contributes to the severity of COVID-19 disease in men and women; however, it has been hypothesised that stronger immune responses in women contribute to their reduced mortality [9].

We evaluated gender differences in terms of inflammatory markers in patients with severe COVID-19 pneumonia. While no difference was observed between male and female participants in terms of age, white blood cell count, lymphocyte count, RDW, AST, CRP, troponin, albumin and D-dimer values, duration of hospital stay, percentage of PNL and haemoglobin, ALT and ferritin values were higher in males. This is in agreement with many studies, where no gender difference has been found between participants in terms of elevated CRP, and the increased ferritin observed in the male participants, which is an important marker of a cytokine storm, was also similar to the literature.

In a study of 781 men and women hospitalised with COVID-19 infection, men were observed to have higher levels of inflammatory markers. Of these, 453 (58%) were male and 328 (42%) were female, and they were of similar age and had similar body mass indices. The men had higher initial CRP, ferritin and IL-6 levels and peak CRP, procalcitonin, ferritin and IL-6 levels compared to women. There was no difference between the men and women in terms of a combination of hospitalisation, death and admission to the intensive care unit [10].

In another study conducted with 876 male and 876 female patients, the male patients with more severe COVID-19 infection had higher CRP, troponin, transaminases and ferritin values and more lymphocytopenia and thrombocytopenia. Systemic inflammatory response syndrome, bilateral pneumonia, respiratory failure and renal failure were significantly more common in males [11].

Of 548 COVID-19 patients included in a study conducted in Wuhan, 279 (50.9%) were male and 269 (49.1%) were female. The men had a higher death rate than the women and higher IL-10, tumour necrosis factor-α, lactate dehydrogenase, ferritin and CRP values, with lower lymphocyte counts observed in the females [12].

Preliminary data show an association between comorbidities, such as chronic pulmonary disease, hypertension and cardiovascular disease, and the severity of COVID-19 [13], and these comorbidities are more common in men than in women [14]. Although COPD prevalence in COVID-19 cases was low in current reports, COVID-19 infection was associated with substantial severity and mortality rates in COPD. Compared to former and never smokers, current smokers were at greater risk of severe complications and higher mortality rate [15]. In our study, COPD was more common in men than in women. We think that this result is related to smoking rates being higher for men than for women worldwide.

Serum transaminase level is generally lower in women than in men, partly due to differences in the fat/muscle ratio, lipid metabolism and hormonal effects on liver cells [16-18]. In a study of 168 patients with severe COVID-19, significantly higher ALT and AST levels were reported in men compared to women [19]. In our study, no significant difference was observed between the genders in terms of AST, but it was observed that AST levels were higher in males, which is in agreement with the literature.

Thrombotic diathesis is common in patients with severe COVID-19 [20], and COVID-19 patients with thrombotic complications generally show a more aggressive disease course. It has been shown that increased D-dimer and decreased platelet count are poor prognostic factors [4,21,22]. In studies conducted with COVID-19 patients with coagulation dysfunction, the patient population was predominantly male, which probably reflects the more severe disease in males [23, 24]. Although the underlying mechanism of coagulopathy in COVID-19 patients has not yet been clarified, it is assumed that a disproportionate inflammatory response leads to endothelial cell dysfunction and a prothrombotic state [25]. It is thought that SARS-CoV-2 may cause endotheliitis due to ACE2 receptor expression in endothelial cells. The consequences of endotheliitis include extensive organ involvement, sudden vasoconstriction, abnormal angiogenesis, microthrombus formation and ischemia [26]. Studies of coagulation factors in the general population have consistently shown more favourable profiles for female subjects, especially younger women of premenopausal age [27]. In our study, while no difference was observed between male and female participants in terms of D-dimer values, thrombocytopenia was found at a higher rate in male patients, in agreement with the literature.

The main limitations of this study are its retrospective design and the fact that it was conducted in a single center. In addition, the immunological markers of patients that have not been evaluated is another limitation.

## Conclusions

Due to the retrospective and single-centred nature of our study, the small number of patients was its limitation. Larger studies with gender-specific reporting and robust analyses are required to clarify how gender alters the cellular and molecular pathways associated with SARS-CoV-2. This would improve the interpretation of biomarkers and the clinical management of COVID-19 patients by facilitating a personalised medical approach to risk stratification, prevention and treatment.
